# Mechanistic Perspectives on Herpes Simplex Virus Inhibition by Phenolic Acids and Tannins: Interference with the Herpesvirus Life Cycle

**DOI:** 10.3390/ijms26135932

**Published:** 2025-06-20

**Authors:** Sherif T. S. Hassan

**Affiliations:** Department of Applied Ecology, Faculty of Environmental Sciences, Czech University of Life Sciences Prague, Kamýcká 129, 165 00 Prague, Czech Republic; sherif.hassan@seznam.cz

**Keywords:** acyclovir resistance, antiviral mechanisms, herpes simplex virus, HSV latency, herpesvirus infections, natural antivirals, phenolic acids, polyphenols, synergistic antiviral effects, tannins

## Abstract

Herpes simplex virus (HSV) is a prevalent and persistent human pathogen belonging to the family *Herpesviridae* and classified as an alpha-herpesvirus. It comprises two distinct types, HSV-1 and HSV-2, which together infect a significant portion of the global population and pose substantial public health challenges. HSV-1 is typically associated with oral herpes, while HSV-2 primarily causes genital herpes; both are characterized by recurrent lesions, latent infection, and mucocutaneous discomfort. Conventional antiviral drugs such as acyclovir and its derivatives are limited by drug resistance, potential toxicity, and their inability to eradicate latent viral reservoirs. These limitations have prompted increasing interest in alternative therapeutic strategies. Phenolic acids and tannins, plant-derived polyphenolic compounds, have attracted considerable attention due to their potent antiviral properties against various viruses, including HSV. This review summarizes current research on phenolic acids and tannins as promising natural antivirals against HSV, with a focus on their mechanisms of action and efficacy in disrupting multiple stages of the HSV life cycle.

## 1. Introduction

Herpes simplex virus (HSV), a member of the *Herpesviridae* family, is a large, enveloped DNA virus capable of establishing lifelong infections in humans. HSV, classified as an alpha-herpesvirus, comprises two distinct types: HSV-1 and HSV-2 [1,2]. HSV-1 is traditionally associated with orofacial infections, including cold sores and herpetic gingivostomatitis, and is a common cause of herpes simplex keratitis and sporadic viral encephalitis [3,4,5]. HSV-2 is primarily responsible for genital herpes, a sexually transmitted infection characterized by painful genital ulcers, systemic symptoms, and severe complications in neonates born to infected mothers [6,7]. Notably, HSV-2 infection significantly increases the risk of acquiring human immunodeficiency virus (HIV) [8,9].

HSV infections can range from asymptomatic or mild to severe and recurrent. Both HSV-1 and HSV-2 establish latency in sensory neurons, with periodic reactivation leading to recurrent lesions [10,11]. While recurrences are generally self-limiting in immunocompetent individuals, they can cause life-threatening, disseminated disease in neonates, individuals with HIV, and patients receiving immunosuppressive therapy [12,13]. In addition to physical symptoms, recurrent HSV infections impose a substantial psychological and social burden, contributing to emotional distress, stigma, and reduced quality of life [14,15].

Current treatment options primarily rely on antiviral drugs such as acyclovir (ACV), valacyclovir, and famciclovir. Although effective in controlling acute episodes and reducing viral shedding, these drugs do not eliminate latent virus, are associated with side effects, and may lead to drug resistance. These limitations underscore the urgent need for alternative therapies [16,17]. Plant-derived compounds have gained increasing attention due to their structural diversity and broad-spectrum antiviral activities. These agents can interfere with multiple stages of the HSV life cycle, including viral attachment, entry, replication, and assembly, thereby lowering the risk of resistance [18]. Among them, phenolic acids and tannins, two major classes of natural polyphenols, have emerged as promising candidates for antiviral drug development due to their diverse biological activities and low toxicity profiles [19].

This review critically examines phenolic acids and tannins as potential natural antiviral agents against HSV. It explores their effectiveness in targeting multiple stages of the HSV life cycle and elucidates their underlying mechanisms of action. By evaluating both preclinical and clinical evidence, the review assesses the therapeutic potential of these polyphenolic compounds and highlights their promise in overcoming the limitations of current antiviral therapies for HSV infections.

The literature search was conducted using several online databases, including the Web of Science Core Collection, Scopus, PubMed, SciFinder, Google Scholar, ScienceDirect, and ClinicalTrials.gov, to collect relevant studies. Appropriate keywords and keyword combinations were used to identify research on the antiviral properties of phenolic acids and tannins against HSV-1 and HSV-2 infections. These keywords included HSV-1, HSV-2, herpesvirus, phenolic acids, tannins, polyphenols, antiviral activity, viral inhibition, viral life cycle, mechanism of action, molecular mechanism, cellular mechanism, natural compounds, plant-derived antivirals, and phytochemicals. The search was particularly focused on studies describing the molecular and cellular mechanisms by which these compounds interfere with the HSV life cycle. The search included studies published between 2017 and April 2025; however, a select number of earlier studies (published before 2017) were also involved to provide historical context and support the interpretation of more recent findings. Inclusion criteria were (i) original research articles or clinical studies reporting the antiviral activity of phenolic acids and/or tannins specifically against HSV-1 or HSV-2; (ii) studies describing molecular or cellular mechanisms of action; and (iii) publications in English. Exclusion criteria comprised (i) review articles; (ii) studies not focused on HSV; (iii) articles lacking mechanistic insights or specific compound information; (iv) non-peer-reviewed sources, editorials, and conference abstracts; and (v) duplicate records across databases.

## 2. Virology and Life Cycle of HSV

### 2.1. Structure and Genomic Organization

Herpes simplex viruses, comprising HSV-1 and HSV-2, are large, enveloped viruses classified under the *Herpesviridae* family, subfamily *Alphaherpesvirinae* [20,21]. They possess a linear, double-stranded DNA genome of approximately 152 kilobase pairs (kbps) that encodes over 80 proteins, many of which are essential for viral replication, immune evasion, and latency establishment [22,23,24].

The HSV virion is composed of four distinct structural components that collectively facilitate viral infectivity and immune evasion. At its core lies the viral double-stranded DNA genome in an uncondensed form [25,26]. This core is enclosed within an icosahedral capsid primarily made of the major capsid protein VP5, comprising 162 capsomers that protect the genome and facilitate its delivery into the host nucleus [27,28]. Surrounding the capsid is the tegument, an amorphous proteinaceous layer containing key regulatory proteins such as VP16 (α-trans-inducing factor) and the virion host shutoff (VHS) protein. These tegument proteins play critical roles in modulating host cellular responses, initiating immediate-early (IE) gene expression, and counteracting host antiviral defenses [29,30,31,32]. Enclosing the entire structure is a lipid envelope derived from host cell membranes, studded with essential viral glycoproteins, such as gB, gC, gD, gH, and gL, which mediate host cell attachment, membrane fusion, and immune system evasion [33,34].

### 2.2. Life Cycle

**Figure 1:**
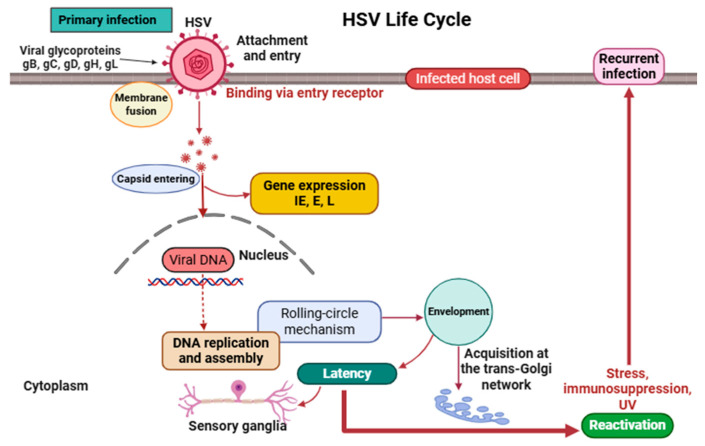
An overview of the HSV life cycle. IE, immediate-early gene; E, early gene; L, late gene.

The life cycle of HSV (Figure 1) includes both lytic and latent phases, comprising a series of tightly coordinated events essential for viral propagation and persistence [35,36]. Infection begins with the binding of viral glycoproteins gB and gC to heparan sulfate proteoglycans on the host cell surface, facilitating initial attachment. This is followed by the interaction of glycoprotein gD with specific entry receptors such as nectin-1 or herpesvirus entry mediator (HVEM), triggering membrane fusion and the delivery of the capsid and tegument proteins into the cytoplasm [37,38,39,40].

The capsid is then transported via dynein motors along microtubules to the nuclear pore, where it releases viral DNA into the nucleus. Gene expression follows a temporal cascade, beginning with immediate-early (IE) genes (e.g., *ICP0*, *ICP4*, *ICP27*, and *ICP47*), which regulate host defenses and viral transcription; followed by early (E) genes (e.g., *UL9* [origin-binding protein], *UL30* [DNA polymerase]) involved in DNA replication; and concluding with late (L) genes encoding structural proteins [41,42,43,44].

HSV replicates its DNA via a rolling-circle mechanism, generating concatemers that are cleaved and packaged into capsids within the nucleus [45]. Capsids transiently acquire an envelope at the inner nuclear membrane, lose it during cytoplasmic transit, and receive their final envelope at the trans-Golgi network before being released via exocytosis [46,47,48,49]. A distinctive feature of HSV is its ability to establish latency in sensory ganglia, where its genome remains episomal and transcriptionally silent except for latency-associated transcripts (LATs), which help maintain latency and inhibit apoptosis [50,51,52,53,54]. Upon exposure to stimuli such as stress, immunosuppression, ultraviolet light, or tissue injury, the virus can reactivate, leading to renewed replication and recurrence of lesions at peripheral sites [55,56,57].

## 3. Overview of Phenolic Acids and Tannins: Chemistry and Antiviral Capacities

Phenolic acids and tannins, two widely distributed plant-derived polyphenols, exhibit significant antiviral properties through distinct yet complementary mechanisms [58]. Phenolic acids are characterized by an aromatic ring bearing one or more hydroxyl groups and a carboxylic acid functional group. They are commonly found in fruits, vegetables, and beverages such as coffee, tea, berries, and whole grains. Chemically, phenolic acids can be broadly divided into two categories: hydroxybenzoic acids (e.g., gallic acid, protocatechuic acid) and hydroxycinnamic acids (e.g., caffeic acid, ferulic acid, p-coumaric acid). The antiviral properties of these compounds are largely influenced by the number and position of hydroxyl or methoxy groups on the aromatic ring, which modulate their redox activity, membrane permeability, and protein-binding affinity [59,60,61]. Phenolic acids exert antiviral effects via several mechanisms. They may directly inactivate free viral particles by altering capsid or envelope integrity through oxidative stress or by interacting with viral surface proteins. Others interfere with viral replication by inhibiting viral DNA polymerase or helicase activity. Moreover, these compounds have been shown to modulate host immune responses—upregulating antiviral cytokines such as interferons and interleukins—thereby enhancing innate defenses against viral infection. Their ability to exert pleiotropic effects across different stages of the viral life cycle reduces the probability of resistance development, which is a key limitation of conventional monotherapies [62,63,64].

Tannins, by contrast, are larger polyphenolic compounds with molecular weights often exceeding 500 Da. They are classified into two major groups: hydrolyzable tannins (such as gallotannins and ellagitannins, composed of gallic or ellagic acid esters) and condensed tannins (also known as proanthocyanidins, consisting of flavan-3-ol oligomers and polymers). Tannins are abundant in the bark of oak and chestnut trees, as well as in grapes, berries, nuts, and teas [65,66,67,68]. Their antiviral mechanisms are multifaceted: tannins can bind directly to viral surface proteins, such as glycoproteins gB and gD in HSV, thereby preventing viral entry into host cells. They also interfere with viral enzymes essential for replication and suppress inflammatory responses by modulating signaling pathways such as nuclear factor-κB (NF-κB) and mitogen-activated protein kinases (MAPKs) [69,70]. The high molecular complexity of tannins enables multivalent interactions with viral targets, often resulting in irreversible viral aggregation or inactivation [71,72]. However, their large size and low aqueous solubility may limit bioavailability, necessitating advanced delivery systems such as nano-formulations or hydrogels to optimize therapeutic efficacy [73,74].

In addition to their direct antiviral effects, phenolic acids and tannins possess strong antioxidant activity, which can help protect host tissues from virus-induced oxidative damage and inflammation [75,76,77].

The multifunctional antiviral actions of phenolic acids and tannins, combined with their natural abundance, structural diversity, and low toxicity, underscore their potential as promising candidates for the development of novel antiviral therapies. Importantly, their synergy with existing antiviral drugs offers opportunities for combination therapies that could enhance clinical outcomes, reduce resistance emergence, and improve tolerability [63,78].

## 4. Anti-HSV Properties of Phenolic Acids and Their Mechanisms of Action

Phenolic acids have garnered considerable interest as potential antiviral agents, particularly for the treatment of HSV infections. Understanding the mechanisms underlying their anti-HSV activity is essential for evaluating their therapeutic efficacy. These mechanisms have been extensively investigated through in vitro, in vivo, and in silico studies. Mechanistic analyses have revealed multiple pathways through which phenolic acids interfere with the HSV life cycle, including direct virucidal effects, inhibition of viral DNA replication, suppression of key viral protein synthesis, and modulation of host antiviral immune responses. For instance, protocatechuic acid, derived from *Hibiscus sabdariffa* L., has been shown to inhibit HSV-2 infection by targeting viral DNA replication in vitro [79]. Its catechol structure (ortho-dihydroxybenzene ring) is thought to contribute to redox-based disruption of viral nucleic acid synthesis. This compound not only inhibits HSV-2 proliferation but may also act synergistically with immune mediators, making it a promising candidate for topical or systemic antiviral development.

Ginkgolic acid, a bioactive compound from *Ginkgo biloba*, exhibits potent anti-HSV-1 effects. In vitro studies demonstrate its ability to suppress viral replication by disrupting the synthesis of critical viral proteins, including ICP27 and US11, which are essential for viral genome replication and immune evasion [80]. Furthermore, ginkgolic acid impairs the formation of new virions, suggesting post-entry interference. In combination studies, *Ginkgo biloba* extracts containing <5 ppm of ginkgolic acid showed broad-spectrum inhibition of both HSV-1 and HSV-2 by blocking viral attachment and entry [81]. Importantly, in vivo experiments using BALB/cJ mice have revealed that topical application of ginkgolic acid reduces mortality, lesion severity, and viral titers in HSV-1 skin infections. Notably, it retains activity against ACV-resistant HSV-1 strains, highlighting its potential as an alternative therapeutic for drug-resistant infections [82].

A polyphenol-rich extract from *Solanum melongena* L. containing caffeic acid, vanillic acid, and chlorogenic acid has demonstrated strong antiviral effects against HSV-1 in Vero cells. These compounds significantly inhibit viral gB expression, a key factor in viral entry and cell fusion, across different phases of infection [83]. Caffeic acid, in particular, exhibits antiviral activity through multiple mechanisms, including chelation with Fe^3+^ ions and interaction with anionic viral proteins. These interactions impair viral attachment, entry, and DNA replication. In addition, metal chelates of caffeic acid have shown improved efficacy compared to the free compound, underscoring their potential as adjuncts to standard antiviral regimens such as ACV [84].

Innovative drug delivery strategies have further enhanced the antiviral effects of phenolic acids. A nano-formulation combining ellagic acid with functionalized zinc oxide nanoparticles (ZnO NPs) has been shown to selectively inhibit HSV-2 replication with superior efficacy compared to ellagic acid alone [85]. The synergistic interaction between the polyphenol and ZnO NPs facilitates targeted delivery to infected cells and disrupts viral DNA synthesis more effectively, representing a promising platform for next-generation antiviral therapeutics.

Molecular docking studies have identified several phenolic acids—trans-ferulic acid, gentisic acid, vanillic acid, syringic acid, and gallic acid—extracted from *Graptopetalum paraguayense* E. Walther, as potential inhibitors of HSV-1 DNA polymerase [86]. These compounds exhibit strong binding affinities for the enzyme’s catalytic domain, suggesting their ability to block viral DNA replication. Supporting this, gallic acid derived from *Punica granatum* was validated in vitro to effectively reduce HSV-1 replication through inhibition of DNA polymerase activity [87]. This dual evidence from computational and biological models strengthens the case for developing phenolic acid-based polymerase inhibitors.

In a complementary approach, p-coumaric acid, identified in *Phoenix dactylifera* L., was shown through in silico analysis to bind the active site of HSV-1 gD, an essential mediator of viral entry into host cells [88]. By targeting gD, p-coumaric acid may prevent viral fusion and entry, a critical early step in HSV pathogenesis. This finding opens the possibility for entry-inhibiting therapeutics based on structurally optimized cinnamic acid derivatives.

Collectively, these studies demonstrate that phenolic acids exert multifaceted antiviral effects, targeting distinct stages of the HSV life cycle including viral entry, genome replication, and structural protein synthesis. Their low cytotoxicity, wide availability, and capacity to overcome drug resistance underscore their potential in the development of plant-based antivirals and combination regimens with existing therapeutics.

**Figure 2:**
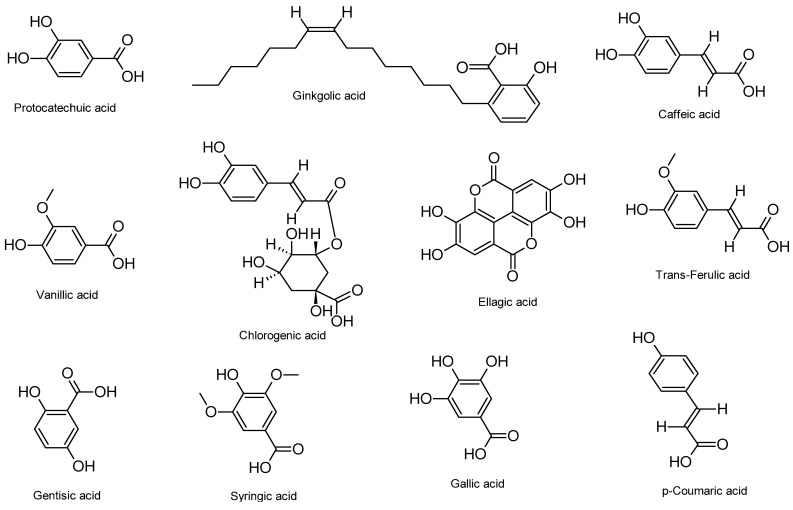
Phenolic acids acting as inhibitors of HSV-1 and HSV-2 infections.

Table 1 summarizes various phenolic acids and their respective mechanisms of action against HSV infections, as identified through in vitro, in vivo, and in silico studies. The chemical structures of these compounds are illustrated in Figure 2.

## 5. Anti-HSV Activities of Tannins and Their Mechanisms of Action

In vitro and computational investigations have provided valuable insights into the anti-HSV mechanisms of tannins. These polyphenolic compounds are known for their complex molecular architectures, which facilitate multivalent interactions with viral proteins, host cell receptors, and intracellular enzymes. Tannins can inhibit multiple stages of the HSV life cycle, including viral attachment, entry, replication, and egress, often through irreversible interference with viral structural components or functional enzymes. Mechanistic evaluations in animal models have further substantiated their efficacy, offering promising avenues for therapeutic development.

Among the best-characterized tannins is geraniin, a hydrolyzable ellagitannin derived from *Spondias mombin* L., which exhibits broad-spectrum antiviral activity. In vitro assays revealed its direct virucidal effect against HSV-1 by disrupting viral particles and blocking their attachment to host cell membranes. Additionally, molecular docking studies demonstrated that geraniin binds effectively to HSV-1 gB [89]. This dual mechanism—virion inactivation and receptor interference—underscores geraniin’s promise as a prophylactic or topical antiviral agent.

Chebulagic and chebulinic acids, isolated from *Terminalia chebula* Retz., have been shown to inhibit HSV-2 by targeting early stages of infection. These ellagitannins suppressed viral binding and internalization into Vero cells, subsequently inhibiting viral DNA replication [90]. Their mode of action suggests that tannins can effectively mimic the entry-inhibitory mechanisms of monoclonal antibodies or fusion inhibitors, but with added benefits such as antioxidative and anti-inflammatory properties.

Ellagitannins, including epiacutissimin A, epiacutissimin B, acutissimin A, and mongolicain, have demonstrated potent anti-HSV-1 activity in Madin-Darby bovine kidney (MDBK) cells. Their unique structures, characterized by non-nucleoside polyphenol cores and multiple hydroxyl functionalities, enable high-affinity binding to viral glycoproteins, particularly those involved in viral fusion and immune evasion [91]. Unlike conventional antivirals that target viral enzymes, these compounds may act allosterically or sterically to hinder glycoprotein-mediated interactions.

The synergy between tannins and conventional antivirals is exemplified by castalagin and vescalagin, C-glucosidic ellagitannins extracted from *Quercus robur*. When used in combination with ACV, these tannins enhanced the antiviral response against both wild-type and ACV-resistant HSV strains. This potentiation is primarily attributed to their interference with viral DNA replication, which complements the mechanism of nucleoside analogues [92]. Animal model studies further support their in vivo efficacy: castalagin significantly reduced HSV-1 titers in the brains and skin of newborn mice, highlighting its therapeutic relevance in neonatal or encephalitic HSV infections [93].

Another ellagitannin, corilagin, shows neuroprotective and immunomodulatory potential in the context of HSV-1 encephalitis. In both in vitro and in vivo models, corilagin suppressed Toll-like receptor 2 (TLR2) signaling, which is often upregulated during HSV-induced neuroinflammation. By downregulating TLR2 and its downstream cytokines (e.g., tumor necrosis factor-alpha (TNF-α) and interleukin-6 (IL-6)), corilagin mitigates viral-induced neuronal damage and improves survival [94]. This unique mechanism situates corilagin at the intersection of antiviral and anti-inflammatory therapy, making it an attractive candidate for CNS-targeted HSV treatments.

Pentagalloylglucose (PGG), a gallotannin widely found in medicinal plants, exhibits potent anti-HSV-1 activity by inhibiting multiple steps in the viral assembly and release process. Specifically, PGG delays the intracellular transport of viral capsids, impairs nuclear export, and downregulates dynein protein expression, which is essential for cytoplasmic trafficking of viral components [95]. Its effectiveness against ACV-resistant HSV-1 strains reinforces its clinical potential, particularly in drug-refractory cases.

Punicalagin, a major ellagitannin in *Punica granatum*, exerts robust antiviral activity against HSV-2 by targeting the viral protease, as revealed by molecular docking simulations [96]. This direct enzymatic inhibition disrupts the maturation of viral proteins required for replication. Moreover, when co-administered with zinc ions, punicalagin demonstrates enhanced virucidal activity against HSV-1, suggesting a synergistic mechanism involving both protease inhibition and metal ion chelation [97].

Technological advances in tannin delivery have opened new therapeutic avenues. Szymańska et al. [98] developed mucoadhesive hydrogels incorporating tannic acid-modified silver nanoparticles (TA-AgNPs), which demonstrated potent anti-HSV activity in both in vitro and in vivo models. These hydrogels blocked viral attachment through inhibition of gB and gC in HSV-1, and suppressed both attachment and entry in HSV-2. When applied in a murine model of genital HSV-2 infection, these formulations significantly reduced viral transmission and lesion severity. Further validating their utility, Orłowski et al. [99] tested TA-AgNPs in a recurrent HSV-2 vaginal infection model. The treatment not only reduced viral load but also enhanced systemic and mucosal immunity by promoting B-cell activation and the production of virus-specific antibodies. These findings demonstrate the immunoadjuvant potential of tannin-based nano-systems and highlight their dual role as antivirals and immune enhancers.

**Figure 3:**
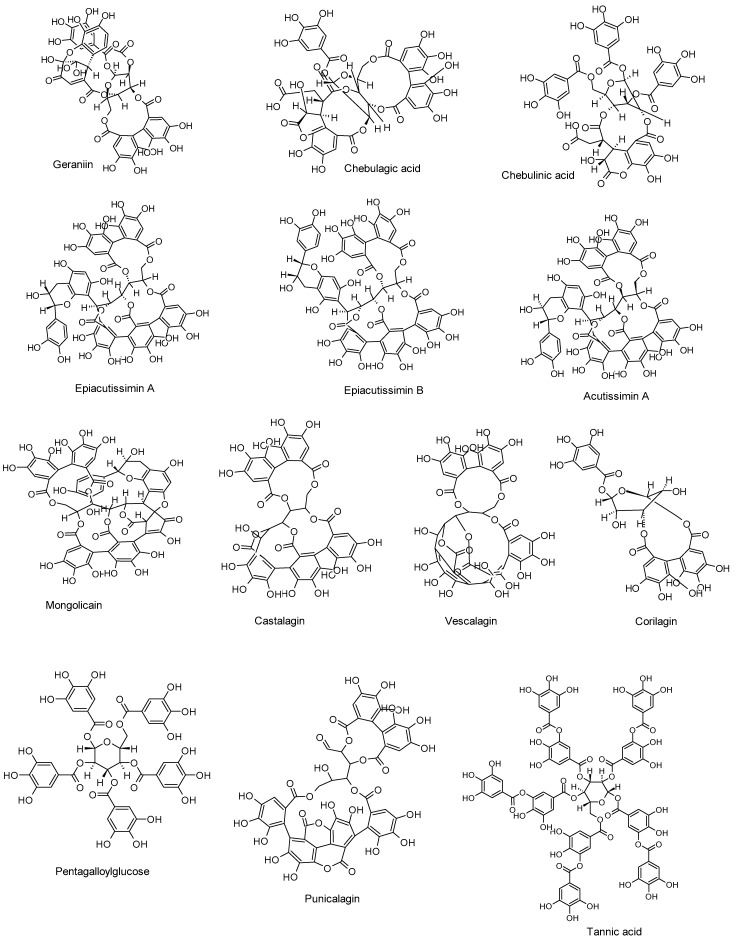
Tannins reported as inhibitors of HSV-1 and HSV-2 infections.

Table 2 outlines the anti-HSV properties of tannins, including the compounds tested, their mechanisms of action, and efficacy as demonstrated through in vitro, in vivo, and in silico investigations. The chemical structures of these compounds are presented in Figure 3.

## 6. Structure–Activity Relationships of Phenolic Acids and Tannins

The antiviral potency of phenolic acids and tannins against HSV is strongly influenced by their chemical structures, particularly the presence and arrangement of hydroxyl, methoxy, and galloyl groups, as well as molecular size and conjugation patterns. Structure–activity relationship analyses provide insight into the functional groups and scaffolds responsible for virucidal, replication-inhibitory, and immunomodulatory actions.

Among phenolic acids, compounds with ortho-dihydroxy configurations, such as protocatechuic acid and caffeic acid, exhibit enhanced redox activity and metal-chelating potential, which are key contributors to their ability to impede viral DNA replication and interfere with HSV glycoprotein function [79,84]. These structural features are also associated with suppression of viral gene expression, as seen with gB downregulation by caffeic acid and related compounds in *Solanum melongena* extract [83]. Conversely, monohydroxylated or methoxylated derivatives such as vanillic and syringic acids tend to display moderate activity [83,86], suggesting that hydrogen-bonding capacity and electron-donating groups are critical to antiviral function.

The presence of esterified or conjugated carboxyl groups, as observed in chlorogenic acid, may facilitate interaction with host or viral proteins, improving membrane permeability and enhancing efficacy at the site of infection [83]. Molecular docking data further support the importance of aromatic hydroxylation for strong binding affinity to HSV DNA polymerase and gD, as demonstrated by gallic acid and p-coumaric acid, respectively [86,87,88].

Tannins, particularly ellagitannins, derive their activity from their large polyphenolic frameworks and high density of hydroxyl substitutions, which enable multivalent binding to viral targets. Compounds such as castalagin and vescalagin feature C-glucosidic linkages that stabilize the molecule and enhance affinity for viral DNA polymerase, especially in drug-resistant HSV strains [92]. The synergy observed when these tannins are combined with ACV is likely driven by their complementary modes of DNA inhibition. In vivo, castalagin has been shown to significantly reduce HSV-1 titers in the brains and skin of newborn mice, underscoring its therapeutic potential in severe infections [93].

Hydrolyzable tannins such as corilagin and punicalagin also demonstrate that specific structural motifs, such as multiple galloyl or hexahydroxydiphenoyl groups, enable interactions with host immune receptors (e.g., TLR2) or viral enzymes (e.g., HSV-2 protease) [94,96]. Gallotannins like PGG, characterized by multiple galloyl units, disrupt capsid transport and nucleocapsid egress, indicating that a higher number of galloyl groups may confer broader functional interference within the viral life cycle [95].

Overall, the antiviral efficacy of these polyphenols appears to be mediated by the following:

- Degree and position of hydroxylation, which affect redox activity and protein binding.
- Conjugation or esterification, influencing solubility and target affinity.
- Multivalency and molecular weight, particularly in tannins, enabling simultaneous interaction with multiple viral or host targets.
- Linkage type and core scaffold stability, which can affect intracellular delivery and resistance to metabolic degradation.

These structural insights provide a rational foundation for future optimization of natural polyphenols as anti-HSV agents, either through semi-synthetic derivatization or formulation strategies.

## 7. Clinical Evidence and Translational Potential

Although much of the evidence for the antiviral properties of phenolic acids and tannins comes from in vitro, in vivo, and in silico studies, emerging clinical findings provide preliminary support for their therapeutic potential against HSV infections. A notable clinical study evaluated a combination therapy involving ultrasound and a topical ointment containing zinc, urea, and tannic acid in 147 women with recurrent genital HSV-2. This treatment significantly reduced lesion-associated pain and accelerated healing time, with a two-year follow-up revealing a substantial reduction in recurrence rates. These findings underscore the potential of tannins, particularly when combined with complementary agents, to enhance the clinical management of HSV infections [100]. The inclusion of zinc and urea in the formulation likely enhanced the dermal penetration and local immunostimulatory effects of tannic acid, supporting the idea that formulation synergy is critical for maximizing therapeutic efficacy.

Despite these encouraging results, clinical validation of phenolic acids and tannins as standalone antivirals remains limited. To date, no large-scale randomized controlled trials have systematically evaluated purified phenolic acids for HSV treatment. Key translational challenges include poor oral bioavailability, rapid metabolic degradation, and a lack of standardized extraction and formulation protocols. Moreover, limited pharmacokinetic and toxicological data on long-term use hinders regulatory approval and broader clinical adoption.

## 8. Conclusions and Future Directions

Phenolic acids and tannins, two structurally diverse classes of plant-derived polyphenols, have demonstrated broad-spectrum antiviral activity against HSV, including strains resistant to standard therapies such as ACV. These compounds act at multiple stages of the viral life cycle—interfering with viral attachment, entry, replication, and sometimes viral egress—and exhibit both direct virucidal effects and indirect immunomodulatory actions. Their ability to interact with key viral enzymes, glycoproteins, and host signaling pathways such as NF-κB and MAPKs supports their multifunctional efficacy.

While laboratory evidence is compelling, a critical limitation remains the paucity of well-controlled clinical trials. To advance these biomolecules from bench to bedside, future research should focus on the design of rigorous placebo-controlled studies that assess not only virological outcomes but also host immune responses, safety, and patient-reported quality of life. Investigating structure–activity relationships may yield derivatives with enhanced potency, while combinatorial studies with conventional antivirals could improve treatment outcomes and reduce resistance risk. Importantly, efforts should be made to enhance the pharmacological profile of these compounds through encapsulation, nano-carriers, mucoadhesive hydrogels, or transdermal delivery systems to improve bioavailability and therapeutic consistency. Furthermore, clinical trials should be designed to include not only classical endpoints such as lesion healing and recurrence but also immunological markers to clarify how these polyphenols interact with host antiviral defenses. If validated through clinical trials and enhanced by pharmaceutical formulation, these compounds could become integral components of next-generation antiviral strategies.

## Figures and Tables

**Table 1:** Phenolic acids with anti-HSV effects across different stages of the viral life cycle.

Compound and Botanical Source	Study Type, HSV Strains, Cells, and Animal Models	Mechanisms of Action (Inhibition)	Effective Concentration/Dose	Refs.
Protocatechuic acid *Hibiscus sabdariffa* L.	In vitro HSV-2 Vero cells	DNA replication	0.9 µg/mL	[79]
Ginkgolic acid *Ginkgo biloba*	In vitro HSV-1 HEp-2, 293T, and Vero cells	DNA replication ICP27, ICP8, US11 protein expressions, viral particles, and post-entry	2.5–50 µM	[80]
	In vitro HSV-1 and HSV-2 A549 cells	Viral attachment, entry, and DNA replication	<5 ppm	[81]
	In vitro and in vivo ACV-resistant HSV-1 Vero cells BALB/cJ mice	Viral particles DNA replication	10 µM (in vitro) 10 mM in 2.5% HEC gel (twice daily for 14 days; in vivo)	[82]
Caffeic acid, vanillic acid, and chlorogenic acid *Solanum melongena* L.	In vitro HSV-1 Vero cells	Viral attachment, entry, DNA replication, and gB expression	IC_50_ = 83.4 µg/mL (the extract)	[83]
Caffeic acid with metal chelates Various plants	In vitro HSV-1 and HSV-2 Vero cells	Viral attachment, entry, and DNA replication	EC_50_ = 27.2 µM (HSV-1) EC_50_ = 17.2 µM (HSV-2)	[84]
Ellagic acid and ellagic acid nano-formulated with ZnO NPs Different medicinal and edible plants	In vitro HSV-2 Vero cells	DNA replication and viral particles	IC_50_ = 4 µg/mL (ellagic acid) and IC_50_ = 3.6 µg/mL (ellagic acid nano-formulation)	[85]
Trans-ferulic acid, gentisic acid, vanillic acid, syringic acid, and gallic acid (*Graptopetalum paraguayense* E. Walther) *Punica granatum* (gallic acid)	In silico HSV-1	Viral replication and DNA polymerase	Binding affinities (118.5–163.4 kcal/mol)	[86]
	In vitro HSV-1 Vero cells	DNA replication	EC_50_ = 10.9 µg/mL (gallic acid)	[87]
p-Coumaric acid *Phoenix dactylifera* L.	In silico HSV-1	gD, viral entry	Binding affinity (not determined)	[88]

Abbreviations: 293T, human epithelial cells; A549 cells, human epithelial cells; ACV, acyclovir; DNA, deoxyribonucleic acid; EC_50_, 50% effective concentration; gB, glycoprotein B; gD, glycoprotein D; HEC, hydroxyethyl cellulose; HEp-2 cells, human epithelial cells; HSV-1, herpes simplex virus type 1; HSV-2, herpes simplex virus type 2; IC_50_, 50% inhibitory concentration; ICP, infected cell protein; US11 protein, an RNA (ribonucleic acid)-binding tegument protein of HSV-1; Vero cells, African green monkey kidney cells; ZnO NPs, zinc oxide nanoparticles.

**Table 2:** Anti-HSV activities of tannins targeting different phases of the viral life cycle.

Compound and Botanical Source	Study Type, HSV Strains, Cells, and Animal Models	Mechanisms of Action (Inhibition)	Effective Concentration/Dose	Refs.
Geraniin *Spondias mombin* L	In vitro and in silico HSV-1 Vero cells	Viral attachment and DNA replication (in vitro) gB expression (in silico)	20.4 µg/mL (in vitro)	[89]
Chebulagic and chebulinic acids *Terminalia chebula* Retz	In vitro HSV-2 Vero cells	Viral attachment, entry, and DNA replication	IC_50_ values of 31.8 and 8.7 µg/mL, respectively	[90]
Epiacutissimin A, epiacutissimin B, acutissimin A, and mongolicain Various medicinal plants	In vitro HSV-1 MDBK cells	DNA replication Viral glycoproteins	16.5–19.7 µM	[91]
Castalagin and vescalagin *Quercus robur*	In vitro HSV-1, HSV-2 (wild types), ACV-resistant HSV-1, and ACV-resistant HSV-2 Vero cells	DNA replication	IC_50_ values ranging from 0.04 to 0.46 µM.	[92]
	In vivo HSV-1 Newborn mice	DNA replication Viral titers	0.02 mL of castalagin (at doses of 7.5 and 10 mg/kg, administered over a 7-day course)	[93]
Corilagin The genus *Phyllanthus*	In vitro and in vivo HSV-1 Vero and BV2 microglia cells Balb/c male mice	DNA replication, TLR2, TNF-α, and IL-6 (in vitro and in vivo)	100 ng/mL (in vitro) 0.4 mg/mouse/day for 5 days (in vivo)	[94]
Pentagalloylglucose Various medicinal plants	In vitro HSV-1 (wild type) and ACV-resistant HSV-1 Vero cells	DNA replication, nuclear transport and nucleocapsid egress, and dynein expression	3.1–10 µM	[95]
Punicalagin *Punica granatum*	In vitro and in silico HSV-2 Vero cells	DNA replication (in vitro) HSV-2 protease (in silico)	31.2 µg/mL (in vitro)	[96]
	In vitro HSV-1 Vero cells	DNA replication	0.05 mg/mL	[97]
Tannic acid with AgNPs Numerous plant sources	In vitro and in vivo HSV-1 and HSV-2 Immortal human keratinocyte cells Murine models	Viral attachment, gB, gC expressions, and DNA replication (HSV-1; in vitro) Viral attachment, entry, and DNA replication (HSV-2; in vitro) HSV-2 vaginal transmission (in vivo)	25 and 50 ppm (in vitro) 25 ppm (in vivo)	[98]
	In vivo HSV-2 Mouse models	DNA replication, viral particles, and viral transmission	5 µg/mouse (administered after 6, 24, and 48 h of infection)	[99]

Abbreviations: ACV, acyclovir; BV2 cells, mouse microglia cells; DNA, deoxyribonucleic acid; dynein protein, a motor protein that plays a crucial role in the transport of viral particles within infected cells; gB, glycoprotein B; gC, glycoprotein C; HSV-1, herpes simplex virus type 1; HSV-2, herpes simplex virus type 2; IC_50_, 50% inhibitory concentration; IL-6, interleukin-6; MDBK cells, Madin-Darbey bovine kidney cells; TA-AgNPs, tannic acid with silver nanoparticles; TLR2, Toll-like receptor 2; TNFα, tumor necrosis factor-α; Vero cells, African green monkey kidney cells.

## Data Availability

As this is a review paper, no original data were generated or analyzed. All information and references used in the review are cited within the manuscript.
